# Rapid green assembly of antimicrobial nanobunches

**DOI:** 10.1038/srep27006

**Published:** 2016-05-27

**Authors:** Jeong Hoon Byeon

**Affiliations:** 1 School of Mechanical Engineering, Yeungnam University, Gyeongsan 38541, Republic of Korea

## Abstract

Antimicrobial nanobunches with different amounts of chitosan-capped Ag were prepared by continuous gas-liquid green route under ultrasound irradiation. Spark-produced aerosol Cu nanoparticles were directly injected into an ultrasound Ag(I)-chitosan reaction cell for efficient hydrosolization of the Cu particles and the subsequent incorporation of Ag and chitosan on Cu. Subsequently, electrospraying was used to form of chitosan-capped Cu-Ag nanobunch coatings. The time required for reducing the bacterial proliferation to 50% dropped to ~1 h at a nanobunch concentration of 10 μg mL^−1^ from the 2.0 min Ag(I) reaction time, and was further decreased to ~0.5 h by increasing the concentration of the nanobunches to 90 μg mL^−1^. The nanobunches were directly coated onto the substrate using an electrospray device to fabricate transparent films and composite fibers. The antimicrobial activity of the composite carbon fibers was then evaluated via the disc diffusion method.

Bacterial contamination of surfaces poses danger to human health and compromises the use of various medical devices and systems^1^. To date, antimicrobial agents, such as silver (Ag), copper (Cu), quaternary ammonium ions, and others, have been used to prevent the growth of harmful bacteria^2^. To increase the use of antimicrobial applications, composite structures containing metallic nanoparticles were recently developed and tested. Metallic particle-containing nanostructures have also been considered as biomaterials (either carriers or vehicles) to deliver therapeutic and diagnostic reagents because of their unique optical and magnetic as well as antimicrobial properties^3^.

Consequently, many have attempted to prepare anisotropic metallic nanostructures because their large surface area enhances the release of antimicrobial components as well as increases the contact area between bacteria and metals^4^,^5^,^6^. Template- or seed-mediated chemical approaches are well known for fabricating anisotropic metallic structures, where preformed ultrafine metallic particles are used as templates or seeds for growing other metallic components on their surfaces. Successive growth of metallic components with unique crystal orientations can introduce various morphologies as hierarchical structures^7^. Wet chemistry preparation methods are developed by using electric potentials, ultrasounds, and high temperature in the presence of surfactants and templates^8^. To use metallic nanostructures in practice, surface coatings and modifications by biopolymers are required for sustainable or controlled antimicrobial activity to inhibit bacterial growth without side effects^9^. However, incorporating metallic nanoparticles and polymeric components for specific purposes is challenging because most metallic nanoparticles are not compatible with polymeric materials, and this limits their use in practice.

Most of the anisotropic nanostructures were prepared by batch wet chemical reduction-oxidation processing in the presence of surfactants and templates to achieve high suspension stability and production yield. To form metallic structures in a chemical bath, a reducing agent (mostly toxic) converts to metal ions to metal atoms; however, this may introduce complexities in the reaction and separation and may have restricted use in biological applications^10^,^11^. Many formulations for biological applications based on wet chemistry involve suspensions of solid particles, which are initially workable for short periods^11^. Moreover, polymeric or organic components with metallic nanoparticles are normally unstable owing to gradual degradation by hydrolysis. Biofunctional nanomaterials in suspension or a colloidal form are therefore not recommended. As a result, the paradigm shift in preparation strategies toward simpler and more versatile processing for fabricating hierarchical nanostructures with antimicrobial properties is currently a challenging research area^12^,^13^.

In this study, the potential use of chitosan-capped Cu-Ag nanobunches to prepare antimicrobial coatings via gas-liquid hybrid chemical route under ultrasound irradiation is discussed. Even though wet chemical approaches have practical limitations in biological applications, alternative strategies are still required to prepare metal-polymer hybrid structures that are continuous, greener, simpler, and more versatile. Indeed, wet chemical approaches have been considered to achieve better selectivity and greater safety in hazardous chemical processes and offer many advantages over conventional batch processes^14^. Specifically, the Ag-chitosan incorporation to incoming Cu nanoparticles from the aerosol to hydrosol route was achieved by transmetallation in an ultrasound Ag(I)-chitosan reaction cell in the absence of toxic reductants and surfactants. Chitosan is nontoxic to mammalian cells (biocompatible), nonantigenic, transparent, and film forming^3^,^15^,^16^,^17^, and is available to conjugate with metallic ions^18^. Because of its cationic surface, chitosan binds the bacteria by the electrostatic interaction between chitosan and bacteria (the bacterial cell walls are negatively charged), and this disrupts the cytoplasmic membrane of the bacteria, thus inhibiting the DNA replication^11^.

In the case of the incorporation of Ag in the liquid state, the hydrated electrons produced during ultrasonic mixing and heating reduce the Ag ions to Ag particles of zero valence. Cu nanoparticles were produced in the gas-phase using spark plasma between Cu electrodes under N_2_ gas flow; the Cu particles were injected into the Ag(I)-chitosan aqueous solution using ultrasound to cap the Cu particles with the newly formed Ag-chitosan (Supplementary Fig. S1). The Cu particles were ultrasonically trapped in the solution when the ultrasound reached the bubbles containing Cu particles in the solution. When solutions containing AgNO_3_ and chitosan were separately injected into the cell with the Cu particles, the Ag ions were reduced and subsequently deposited on the trapped Cu particles by ultrasound-assisted transmetallation and solution mixing, forming chitosan-capped Cu-Ag nanobunches^19^. The process of forming nanobunches as a function of reaction time is shown in Fig. 1. The nanobunches were then atomized via electrospraying to fabricate transparent films and composites fibers, which were then used to inhibit bacterial growth (Supplementary Fig. S1).

## Results and Discussion

A gas temperature of approximately 6000 °C was generated between two cylindrical Cu rods, and parts of the rods evaporated^20^. The frequency of spark formation was 320 Hz, and the evaporated Cu nucleated right after the spark channel by N_2_ gas flow, forming Cu nanoparticles. The particle size distribution was analyzed using a scanning mobility particle sizer (3936, TSI, USA) to verify the concentration, mean diameter, and standard deviation. The measured concentration, diameter, and standard deviation were 3.10 × 10^7^ particles cm^−3^, 14.2 nm, and 1.40, respectively, as shown in supplementary Fig. S2. The spark-produced Cu particles were successively injected into an aqueous Ag(I)-chitosan solution in the presence of ultrasound to cause bubble implosion for efficient collection. To verify the quantitative collection of Cu particles, we measured the size distribution with and without ultrasound. Most incoming Cu particles were trapped (96.8% trapping efficiency) in the aqueous solution during ultrasound irradiation. This may be because the bubbles collapse inside before reaching the surface of the solution, resulting in the hydrosolization of nearly all Cu particles. The spark-produced Cu particles in the gas-phase sampled on a carbon-coated copper grid were analyzed using a transmission electron microscope (TEM, JEM-3010, JEOL, Japan) (Supplementary Fig. S2). The Cu particles were electrostatically deposited on the grid using a commercial aerosol collector (NPC-10, HCT, Korea). As shown in supplementary Fig. S2, the TEM observations suggest that the primary Cu particles (~3.3 nm mean diameter) were agglomerates (~14 nm), implying that the primary particles collided with each other after they formed near the spark.

**Figure 1:**
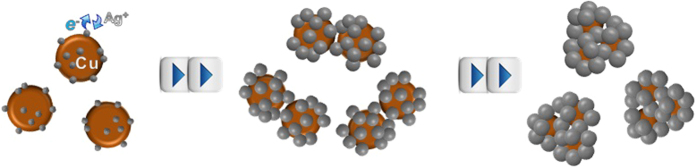
Schematic of the Cu-Ag nanobunch formation with spark-produced Cu nanoparticles continuously injected into the ultrasound Ag(I)-chitosan reaction cell.

**Figure 2:**
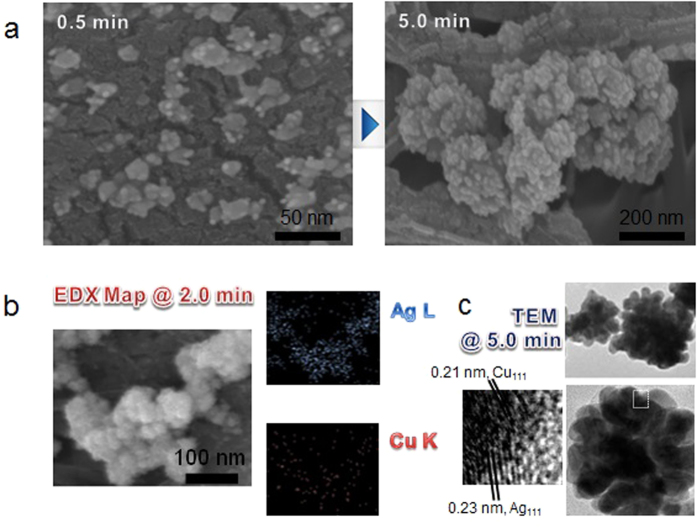
(**a**) SEM images of the growth of Cu-Ag with reaction time with spark-produced Cu nanoparticles continuously injected into the ultrasound Ag(I)-chitosan reaction cell. (**b**) EDX maps and the corresponding SEM images of the nanostructures after 2.0 min reaction time.

The different shapes (from scanning electron microscope (SEM, JSM-6500F, JEOL, Japan) and TEM observations) of the Cu-Ag nanostructures with increasing reaction time were observed to understand the morphological changes during the reaction. For this purpose, particles produced at reaction times 0.5 min, 2.0 min, and 5.0 min were sampled and observed using the SEM to confirm the morphological changes. In the early stage of the Ag(I) reduction, as shown in Fig. 2a, small bunches having different contrasts (i.e., different materials) and sizes were observed. After 5.0 min, these structures grew larger and the continued reaction induced the formation of arbitrarily structured bunches of Cu-Ag (i.e., continuous overlay of Ag on preformed particles). Because of the autocatalysis of the preformed Ag on the trapped Cu particles, larger scale of Ag(I) reduction occurred site-selectively on Ag than the surfaces of the trapped Cu particles (Fig. 1). Figure 2b shows the energy dispersive X-ray (EDX, JED-2300, JEOL, Japan) maps of the samples after 2.0 min of reaction. The SEM images and corresponding elemental maps of Ag and Cu well match each other, indicating the specific locations of Ag and Cu in the SEM images. The TEM results (Fig. 2c) are also consistent with the SEM observations (5.0 min). The TEM images also show different contrasts among the sampled particles, implying that different materials merged to form the particles (Fig. 2c). Sampled particles with darker contrast have the (111) plane owing to the 0.21 nm fcc Cu lattice, whereas the lighter-contrast overlays on the darker ones are attributable to the Ag (111) plane with 0.23 nm lattice spacing. The different parts are combined to form a single body, and it is clearly observed that the Ag deposition on the surfaces of the trapped Cu particles and the preformed Ag was continuous. By increasing the reaction time, the size of the produced structures increased owing to the continuous incoming of Cu and Ag deposition, which eventually produced the larger anisotropic bimetallic structures. The different kinetics of the Ag deposition on the surfaces of the incoming Cu and preformed Ag could maintain the anisotropic Ag formation during the reaction. For comparison purposes, ultrasound-assisted Ag(I) reduction was performed without the incoming Cu particles, and the results suggested that spherical Ag particles were predominantly produced even at the same reaction conditions (Supplementary Fig. S3), which suggests that the incoming Cu into the Ag(I)-chitosan reaction cell was critical to the formation of Cu-Ag nanobunches. The formation of Ag layers was also verified by UV-vis absorption spectroscopy (330, Perkin-Elmer, USA) (Supplementary Fig. S4). The spectra of the nanostructures show that the incorporation of Ag on Cu generates an absorption shift in the visible range. A blue shift from 590 nm to 430 nm in the absorption spectra can be seen via the increase in reaction time (0.5–5.0 min), suggesting that the size and morphology of the structures have changed. The two peaks confirm the existence of Cu and Ag components, respectively. The increase in absorption intensity with increasing reaction time further supports the size increase of the structures. The narrower absorption distribution may have originated from a significantly larger fraction of Ag, which also suggests that the nanostructures are well dispersed.

**Figure 3:**
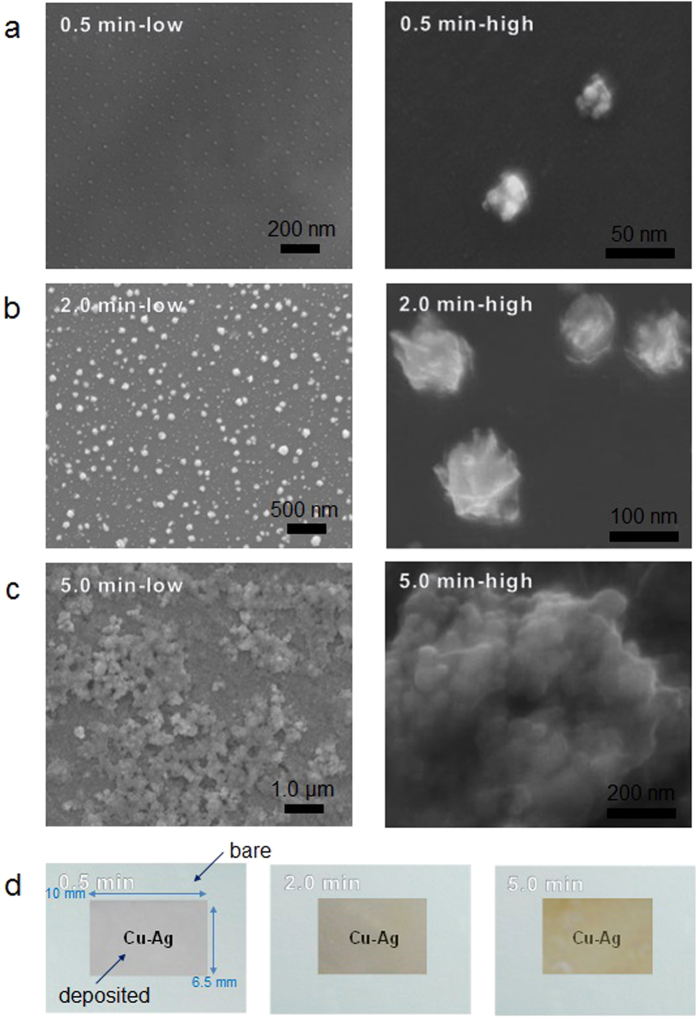
Transparent coating of chitosan-capped Cu-Ag nanobunches. SEM micrographs of the bimetallic nanobunches with reaction time (**a**) 0.5 min, (**b**) 2.0 min, and (**c**) 5.0 min. (**d**) Photos of the nanobunch-deposited glass substrates on “Cu-Ag” printed papers with increasing reaction time.

The synthesized materials were then aerosolized as thin films on a glass plate using an electrospray device. Supplementary Fig. 3a–c show low- and high-magnification SEM images of the electrosprayed chitosan-capped nanobunches on glass plates with increasing reaction time in the Ag(I) cell. When the reaction time was set at 0.5 min, the produced nanostructures were aggregates because of the coalescence between the small products (Fig. 3a). This may be due to the significant increase in diffusion (in the order of 10^3^) when the small particles moved from liquid (hydrosol) to gas (aerosol) by electrospraying the solution, which significantly increases the collision between the products before they reach the substrate. Increasing the reaction time from 2.0 min to 5.0 min caused significant size and shape changes. In the case of 2.0 min reaction time (Fig. 3b), the metallic particles connected with the chitosan matrix had an elongated shape. When the reaction time was 5.0 min (Fig. 3c), larger sized structures were produced and the size deviations increased in comparison with the particles produced with the 2.0 min reaction time. This implies that the larger-sized particles coalesced and created significantly larger aggregated structures during the electrospraying process. Supplementary Fig. S5a shows the EDX maps of the deposited particles. The fraction between Ag and chitosan increased with increasing reaction time. Supplementary Fig. S5b shows the Fourier transform infrared spectra (FTIR, IFS 66/S, Bruker Optics, Germany) of the deposited particles. The results suggest that the nanobunches well conjugated with chitosan because of the binding between Ag and the amino/hydroxyl groups. Fig. 3d shows three glass plates on a “Cu-Ag” printed white paper with a coating of nanobunches electrosprayed on them. Antimicrobial coatings are functional coatings that are not only critical to the general hygiene but also prevent the spreading of infections from physical contact with electronic devices (e.g., touch screen panels); hence, the chitosan-capped nanobunches were further tested for their transparency. As shown in Fig. 3d, the coatings of chitosan-capped Cu-Ag nanobunches have different transparencies with increasing reaction time, and the word “Cu-Ag” was visually verified, implying that the coating was transparent. The thickness of the coating on the glass plate was ~60 μm. The relative opacity (*E*) of the coatings was determined using a handheld chromameter (CL-200A, Konica Minolta Americas Inc., US), and the *E* values of the 0.5 min, 2.0 min, and 5.0 min reaction time cases were 3.55, 10.82, and 25.73, respectively. The corresponding haze values were 1.31%, 3.82%, and 4.66%, respectively. Supplementary Fig. S6 shows other examples for testing transparency, including a mobile touch screen panel.

**Figure 4:**
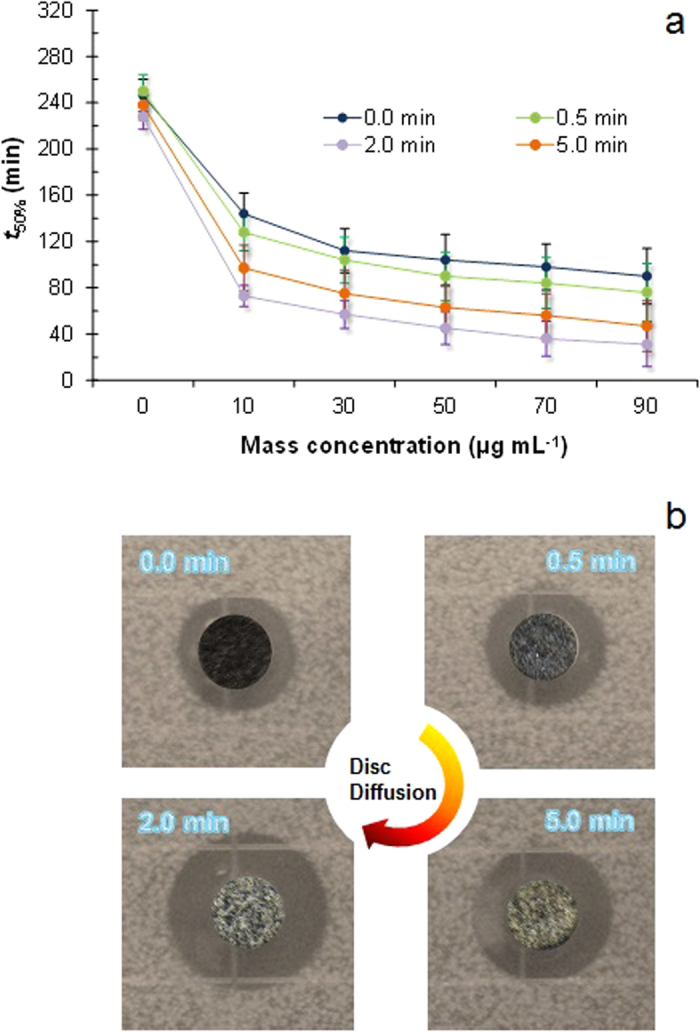
Antimicrobial activity of the chitosan-capped Cu-Ag nanobunches. (**a**) Effect of the reaction time on the fabrication of bimetallic nanobunches regarding the required time to inactivate 50% of the initial bacterial concentration. (**b**) Antimicrobial activity of the nanobunch-deposited carbon fibers with increasing reaction time against *E. coli* as evaluated using the disc diffusion method.

Figure 4a clearly shows that the prepared nanobunches can significantly inhibit the growth of *E. coli* 25922. During the test, water caused chitosan swelling, which may enhance the metallic ion transport to the culture media through the chitosan matrix and inhibit the growth of bacteria. The measured colony-forming units per unit volume of media (cfu mL^−1^) of *E. coli* significantly decreased with increasing nanobunch concentration. After 1.5 h incubation time, less than 50% bacteria were found at the concentration of 90 μg mL^−1^ in all cases. In particular, 50% of the proliferation is inhibited at the concentration of 10 μg mL^−1^ for the 2.0 min reaction time after only ~1 h incubation. The corrosion of the surfaces of the nanobunches might be a reason for the antimicrobial activity because of the water diffusion at the surface of the nanobunches^21^, which supports the antimicrobial behavior. Thus, the smaller sizes of nanobunches for the 2.0 min reaction time were compared to those for the 5.0 min reaction time to determine better antimicrobial activity, because the diffusion of metallic ions (mostly Ag ions) and particles in wet media is inversely proportional to the particle size^22^. This is consistent with the batch growth profiles of these nanobunches (Supplementary Fig. S7). Therefore, it suggests that controlling the reaction time for nanobunch preparation not only inhibits the growth of bacteria but also controls the growth kinetics of the bacteria. The antimicrobial properties of the nanobunches were further tested using the Kirby-Bauer disc diffusion method (Fig. 4b). The inhibition zones around the discs became rather clear and were clearly observed when the nanobunch-deposited carbon fiber disc was placed on the culture media. Furthermore, the susceptibility constant *Z* (mL μg^−1^) of the nanobunches formed at 2.0 min reaction time was defined and compared to that of the spherical Ag nanoparticles (nearly the same mean lateral dimension of ~94 nm with the nanobunches) to further evaluate the antimicrobial properties of the nanobunches against *E. coli*, as shown in supplementary Fig. S8. Briefly, more active antimicrobial materials have high *Z* values, implying that the bacteria have greater sensitivity to these materials. The averaged *Z* values of the bacteria in relation to the Cu-Ag nanobunches and Ag nanoparticles were 0.0745 and 0.0297 mL μg^−1^, respectively. The antimicrobial properties of the chitosan-capped nanobunches (2 min) were further evaluated against *E. coli* and Staphylococcus aureus 25923 in comparison to pure chitosan, Ag, and Cu-Ag samples (10–90 μg mL^−1^, approximately 90 nm lateral dimension) using the minimal inhibitory concentration (MIC) which is standard measurement to determine the bactericidal effects of antimicrobial agents (Supplementary Table S1). The MIC value of chitosan-capped nanobunches was lower than those from pure chitosan, Ag, and Cu-Ag samples, and this implies that the nanobunches were found to be more potent antimicrobial materials. In addition, both antimicrobial activities did not show any significant differences in the two bacterial cells. To support antimicrobial activity, the levels of reactive oxygen species (ROS) were further measured for the nanobunches in comparison to pure chitosan, Ag, and Cu-Ag samples since the ROS analyses are employed as a collective marker for superoxide anion, hydroxyl radical and hydrogen peroxide, which act as an indicator for cellular oxidative stress. The generation of intracellular ROS was determined with 2′,7′-dichlorodihydrofluorescein diacetate by measuring fluorescence intensity as the response of bacterial cells towards the chitosan-capped nanobunches (Supplementary Fig. S10). After 4 h incubation, larger ROS values were detected in the nanobunch treated bacterial cells than other samples, and this implies that cell membrane, protein structure, and/or intracellular system may be more damaged due to larger amounts of ROS for the nanobunch treatments. Therefore, as suggested in the previous reports^23^,^24^, these results support the generation of free radicals for inhibition of bacterial growth.

The cytotoxicity of the nanobunches was further tested because polymer-coated metallic particles are also of interest in biomedicine. The nanobunches were incubated with 293 human embryonic kidney cells for 24 h, and the 3-(4,5-dimethylthiazol-2-yl)-diphenyltetrazolium bromide (MTT) assay was used to evaluate the cell viability in incubation cells with nanobunch samples (Supplementary Fig. S11). The cell viability was approximately 17% at 90 μg mL^−1^ for pure Cu-Ag nanobunches (without chitosan-capping), whereas the cell viability for chitosan-capped Cu-Ag nanobunches was >74% at the same concentration. Though pure Cu-Ag nanobunches released metallic components and clearly contributed to cytotoxicity, the released fraction of the chitosan-capped nanobunches did not affect the cells significantly because of the biocompatible chitosan overlayer. This is consistent with a previous report regarding the decreased cytotoxicity of Ag nanoparticles to mammalian cells because of the protective chitosan overlayers^25^. These results suggested that the chitosan-capped nanobunches not only have an antimicrobial effect on bacteria, but they also have better biocompatibility than pure metallic nanobunches.

A continuous gas-liquid hybrid chemical route under ultrasound irradiation was used for the first time to produce chitosan-capped bimetallic nanobunches and their coatings. The antimicrobial properties and transparency including the biocompatibility of the prepared nanobunches were tested. Electrospraying of the prepared suspension containing Cu-Ag nanobunches allows the fabrication of antimicrobial coatings on the surfaces of different substrates. Continuous single-pass system offers flexibility concerning the combination of the resulting antimicrobial materials and coatings by simply selecting appropriate spark ablation electrodes and biofunctional polymeric/organic components. A further study to optimize the proposed method for the real application regarding thermosensitive antimicrobial properties is now in preparation to publish elsewhere.

## Additional Information

**How to cite this article**: Byeon, J. H. Rapid green assembly of antimicrobial nanobunches. *Sci. Rep.* **6**, 27006; doi: 10.1038/srep27006 (2016).

## Supplementary Material

Supplementary Information
