# Supplementary material for: Rapid green assembly of antimicrobial nanobunches
Source: Sci Rep. 2016 May 27;6:27006. doi: 10.1038/srep27006 (PMC4882583; doi:10.1038/srep27006)
Supplement: Supplementary Information [file srep27006-s1.pdf]

Supplementary Information for Publication

Rapid green assembly of antimicrobial nanobunches

Jeong Hoon Byeon[*]

School of Mechanical Engineering, Yeungnam University, Gyeongsan 38541, Republic of Korea

Experimental procedure

As shown in **Fig. S1**, a spark was generated between two Cu rods (diameter: 3 mm, length: 100 mm, Nilaco, Japan) inside a reactor within an N_2 environment at standard temperature and pressure. The specifications of the spark plasma were as follows: resistance, 0.5 MΩ; capacitance, 1.0 nF; loading current, 1.2 mA; applied voltage, 3.2 kV; and frequency, 320 Hz. The flow rate of the gas was controlled by mass flow controller (Tylan, US), and the flow rate was 3.0 L min^{-1}. The resulting Cu particles were injected into the Ag(I) cell containing chitosan to employ as seeds for synthesizing Cu-Ag nanobunches. In the Ag(I) cell, solutions 1 and 2 were injected with the aid of a peristaltic pump (323Du/MC4, Watson-Marlow Bredel Pump, US) at constant rates of 0.42 and 2.08 mL min^{-1}, respectively. 85 milligrams of $AgNO_3$ (205052, Sigma-Aldrich, US), used as a precursor of Ag, was dissolved in 5 mL of deionized water (Solution 1). 0.25 grams of chitosan (41796, Sigma-Aldrich, US), used both as a reducing agent and a stabilizing agent, was dissolved in 25 mL of 1% acetic acid solution (03888, Sigma-Aldrich, US) (Solution 2). An ultrasound probe (VCX 750, 20 kHz, Sonics & Materials Inc., US) was immersed into the solution. The probe acted as an ultrasound irradiator (10 W mL^{-1} input power density) and the active part of the probe was the planar circular surface, of area 1.3 cm^2, at the bottom of the probe. Upon the start of reactions, the pale yellow solution changed to light brown indicating the reduction of Ag(I). The Cu particles from gas-phase acted as seeds and became kinetically capable of incoming Ag ions onto the seed particles to create Cu-Ag nanobunches. Because the reduction rate of the Ag ions is faster (Ag^+/Ag^0=0.78 V) than that of Cu^{2+}, the reduction of the Ag ion is preferred to that of the Cu particles. Ag layers which are formed on the surface of the Cu particles in the reaction act as the active sites for the further deposition of Ag species.

Chitosan-capped Cu-Ag nanobunches were atomized via electrospray, by means of the equipment assembled in-house. In brief, the equipment consisted of another peristaltic pump (07522-30, Masterflex, US), a stainless-steel nozzle (inner diameter 0.3 mm), a high-voltage

power supply (10/40A, Trek, US), and a stainless steel plate placed directly below the nozzle as the grounded count electrode at a distance of 6 mm between the capillary and the substrate. To find the resultant electrical field required for a stable meniscus, the following equation was employed:

$$E_0 = \left[\frac{2\gamma\cos\theta}{\varepsilon_0 r}\right]^{\frac{1}{2}} \qquad \text{(S1)}$$

where E_0 is the onset electric field, γ is the surface tension of the solution, θ is the angle of the Taylor cone, ε_0 is the permittivity of the vacuum, and r is the radius of the capillary. As shown in **Fig. S1**, the prepared solution forms a stable jet, which is much smaller than the nozzle diameter at a position below the nozzle (monitored with a high-speed camera, Motion Pro, US). The chitosan-capped nanobunch coating after solvent evaporation was formed on a substrate (glass plate or carbon fiber) contacted with a plate heater. The prepared solution was electrosprayed (16 μL min^{-1}) using the pump to perform a coating with chitosan-capped Cu-Ag nanobunches on a glass plate (7059, Corning, US). The electrosprayed nanobunches were also directly deposited on carbon fibers to evaluate their antimicrobial activity via the disc diffusion method.

The bacteria were grown overnight in Luria-Broth at 37°C for the antimicrobial tests. The bacterial count was standardized to ~10^6 cfu mL^{-1} by measuring their optical density and maintained at 37°C for 2 h. The initial concentration of bacteria was determined by the agar plate method. An aliquot of 50 mL of the bacteria broth was poured onto the nanobunch surface and covered with a glass for a specific time ranging from 10 to 300 min at 37°C. After the time elapsed, the samples were washed with a solution of 0.9 wt % of NaCl and 1.0 wt% of Tween solution, an aliquot of 40 μL was extracted, transferred onto a nutrient agar plate and incubated for 16 h at 37°C before the colonies were counted. The minimum inhibitory concentration was also obtained using E. coli and S. aureus in nutrient agar medium after 24 h of incubation.

The generation of intracelluar reactive oxygen species (ROS) generation in nanobunch treated bacterial cells was determined using a cell-permeant fluorescent dye 2',7'-dichlorodihydrofluorescein diacetate (H_2DCFDA), an intracellular ROS indicator.[S1]

All experiments were performed in triplicate, and the results were reported as average values and standard deviations.

FIG. S1

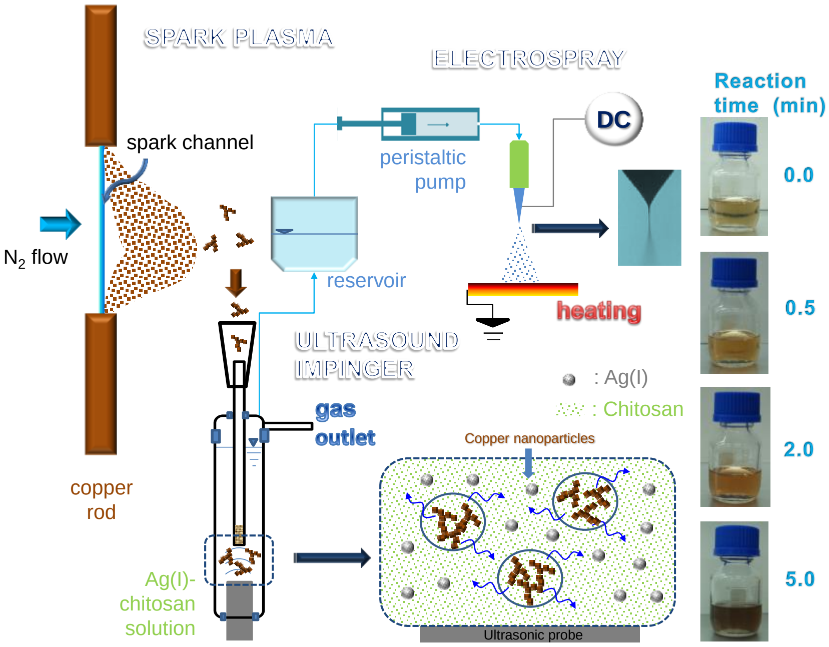

Schematic diagram of continuous gas-liquid hybrid chemical route to fabricate chitosan-capped Cu-Ag nanobunches through a serial system consisting of a spark plasma, an ultrasound Ag(I) cell, and an electrospray device with a heated ground plate

FIG. S2

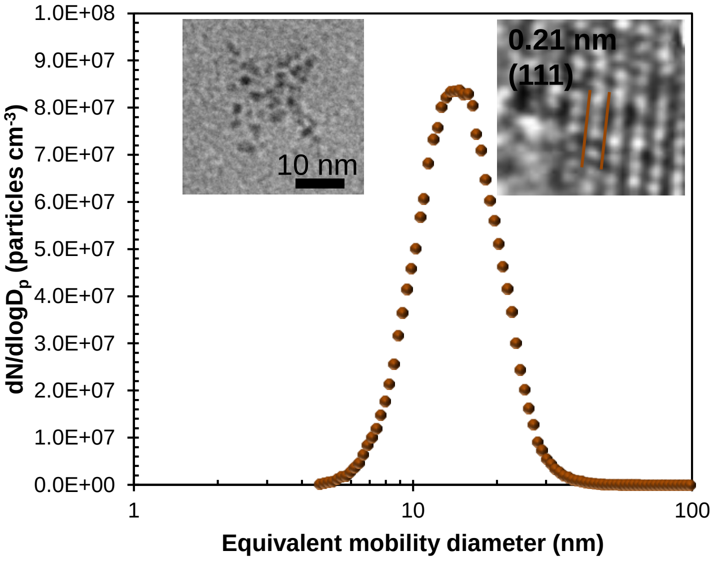

A size distribution of the spark-produced Cu nanoparticles. Representative TEM images of the Cu particles are also shown as insets.

In the case of pure Cu colloidal solution, 0.1 mL of the purified solution was dropped on the TEM grid, and then dried to remove solvent from the drop on the grid for scanning SEM and TEM observations. According to the inset in **Fig. S2**, the most frequently observed lattice spacing was approximately 0.21 nm, which can be matched to the (111) plane of a face-centered cubic (fcc) Cu structure.[S2]

FIG. S3

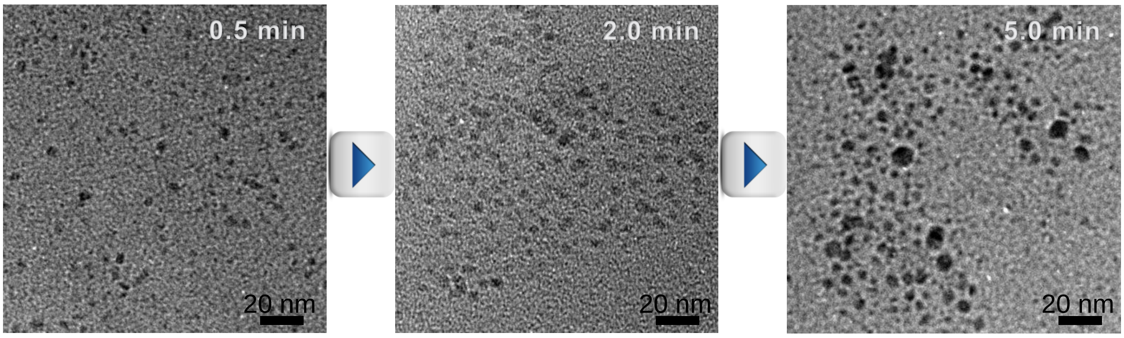

TEM images of Ag nanoparticles with increasing reaction time synthesized in the absence of Cu particles.

FIG. S4

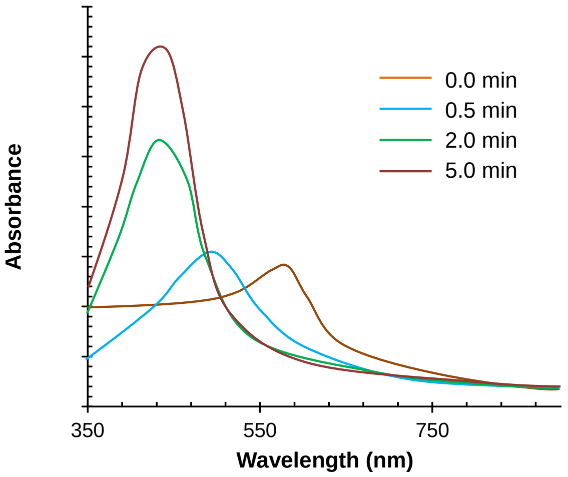

UV-vis spectra of Cu-Ag nanobunches with increasing reaction time ranging from 0.5-5.0 min in the Ag(I) cell.

FIG. S5

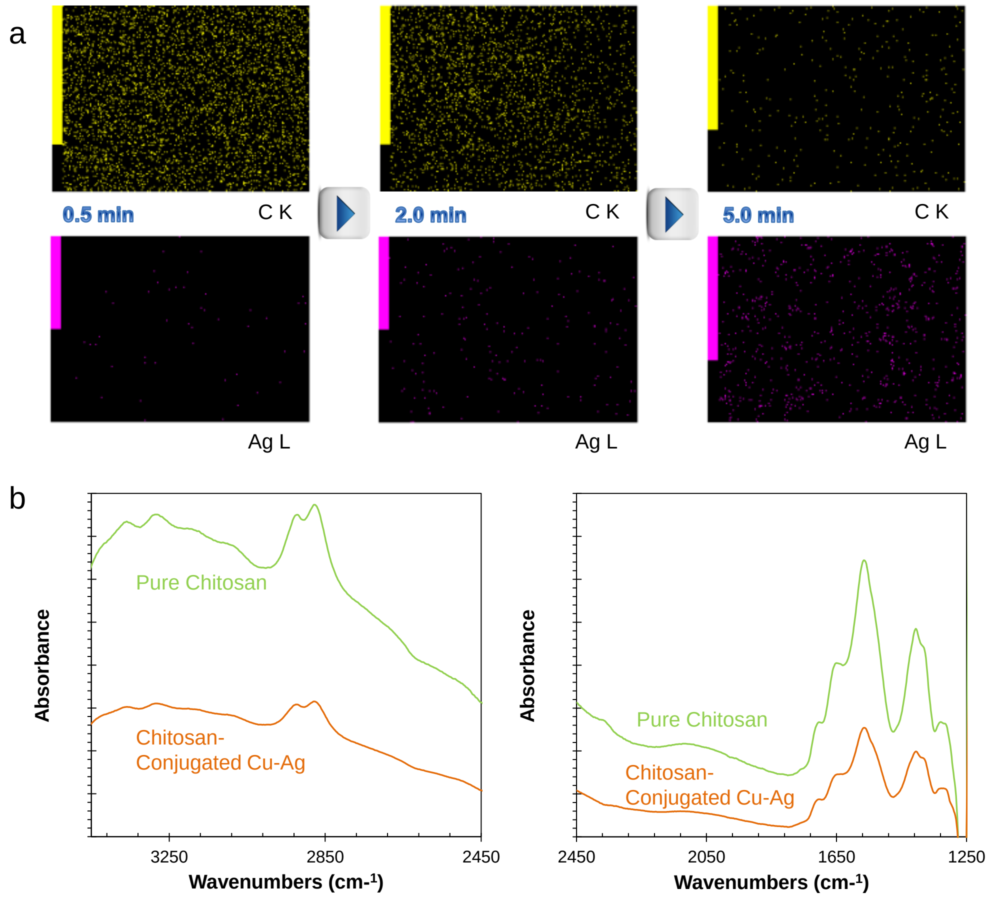

(a) EDX maps of electrosprayed particles with increasing reaction time ranging 0.5-5.0 min. (b) FTIR spectra of electrosprayed pure chitosan nanoparticles and chitosan-conjugated Cu-Ag nanobunches (5.0 min reaction time).

The stretching vibrations of N-H and O-H were assigned to the 3,200 cm^{-1} and 3,400 cm^{-1} broad peaks, respectively, and the bands at 2,940 cm^{-1} and 2,960 cm^{-1} were attributed to the CH_2- and CH_3-bends, respectively. The peaks around 1,580 cm^{-1} corresponding to the N-H bending vibration[S3] moved to 1,570 cm^{-1} and their intensity decreased. This suggests that the Ag atoms obtained from Ag ions in the presence of chitosan could be conjugated with nitrogen atoms during ultrasonification. Thus, the vibration intensity of the N-H bond could be reduced by increasing the molecular weight owing to the Ag conjugation.[S4] In addition, the peak at around 1,660 cm^{-1} corresponds to the N-H scissoring from the primary amine groups that are being dissociated, and the intensity decreases at around 3,420 cm^{-1} of the O-H stretching from the hydroxyl were identified when the Ag atoms were capped by chitosan molecules, suggesting that Ag atoms were conjugated with the amino and hydroxyl groups of chitosan, as shown in **Fig. S5**b.

FIG. S6

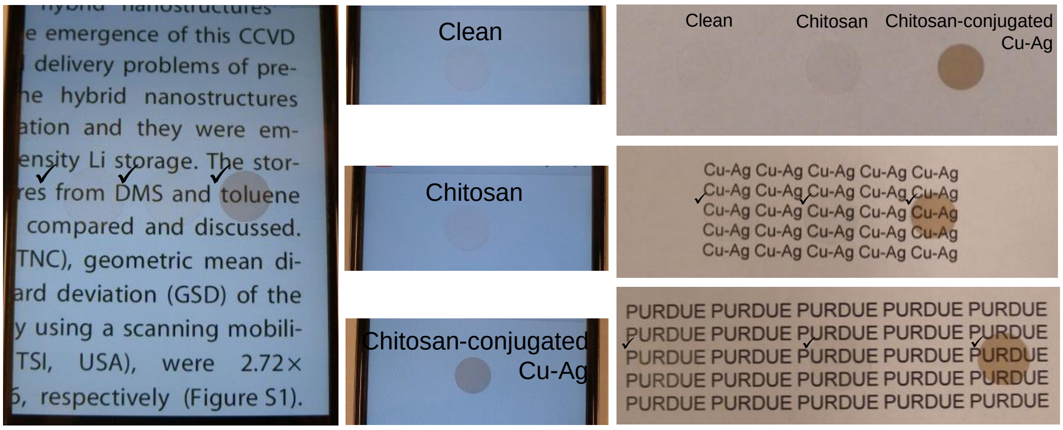

Photographs of glass discs as untreated, chitosan-coated, and chitosan-capped Cu-Ag nanobunch-coated on a mobile display device, and on white and printed papers.

The figure shows that the nanobunch-coated glass plate is transparent, although there are differences in the transparency among the bare, chitosan-coated, and the nanobunch-coated glass plates.

FIG. S7

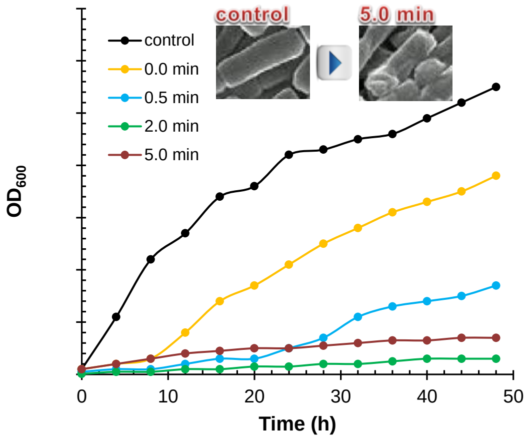

Representative batch growth profiles in the presence of chitosan-capped Cu-Ag nanobunches with increasing reaction time ranging from 0.5-5.0 min. SEM images of *E. coli* before and after treatment with the Cu-Ag nanobunches (2.0 min reaction time) are also shown as insets.

FIG. S8

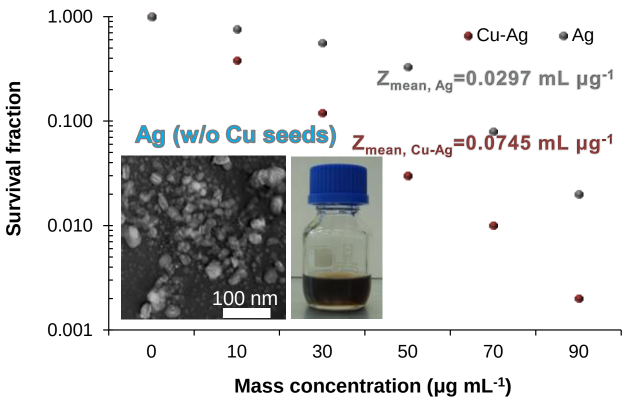

Antimicrobial results of purified spherical Ag nanoparticles and Cu-Ag nanobunches with different mass concentrations (0-90 μg mL^{-1}). A SEM image and photo of the Ag particles are also shown as insets.

The high antimicrobial activity of nanobunches corresponds to the efficient diffusion of the metallic ions, which introduces more significant inactivation of DNA and relevant cellular components that subsequently generates pits in the bacterial cell walls, resulting in enhanced diffusion into the cells (inset of **Fig. S7**).[S5] The wrinkled membranes on the nanobunch-treated cells verify the ability of the nanobunches to disrupt the membrane. A larger number of metallic ion diffusion points of the nanobunches (**Fig. S9**) owing to the smaller primary metallic particle sizes and anisotropic overall shapes might induce higher antimicrobial activity compared to the spherical Ag particles.

FIG. S9

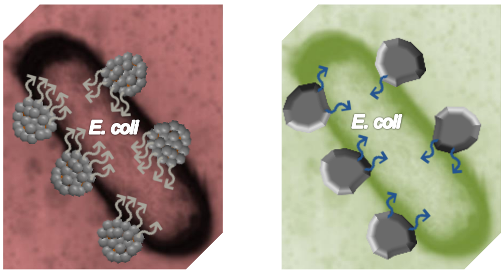

Different antimicrobial activities with spherical Ag nanoparticles and Cu-Ag nanobunches toward *E. coli*. A larger number of bumps (favorable for diffusing Ag ions onto cell membranes) on the nanobunches than those on the Ag nanoparticles might be more suitable for efficient inactivation of cell proliferation.

FIG. S10

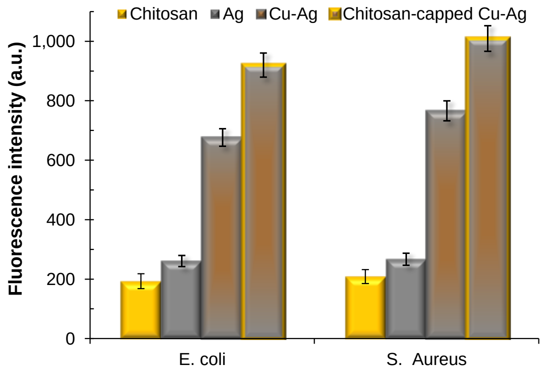

Generation of intracellular ROS in the bacterial cell suspensions treated with chitosan-capped nanobunches (2 min) including pure chitosan, Ag, and Cu-Ag samples (at 50 μg mL^{-1} for 4 h incubation).

FIG. S11

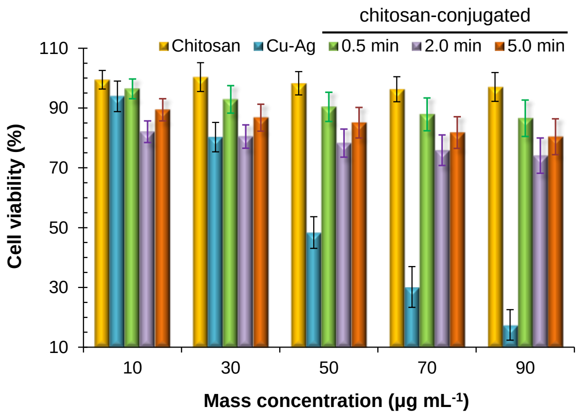

Cytotoxicity of chitosan nanoparticles, and purified and chitosan-capped Cu-Ag nanobunches with different mass concentrations (0-90 μg mL^{-1}) in HEK 293 cells.

Chitosan provided a higher affinity than the nanobunches themselves with the negatively charged biological membranes. This suggests that the chitosan-capped nanobunches are not cytotoxic and are likely suitable as antimicrobial coatings of biomedical devices.

SUPPLEMENTARY TABLE

Table S1 MIC values of chitosan-capped nanobunches in comparison to pure chitosan, Ag, and Cu-Ag samples

	MIC (μg mL^{-1})			
Bacterial cell	Chitosan	Ag	Cu-Ag	Chitosan-capped Cu-Ag
E. coli	50.1	45.8	16.5	8.6
S. aureus	52.2	45.8	17.3	9.9

SUPPLEMENTARY REFERENCES

S1. Choi, O. & Hu, Z. Size dependent and reactive oxygen species related nanosilver toxicity to nitrifying bacteria. *Environ. Sci. Technol.* **42**, 4583-4588 (2008).

S2. Jia, X. & Wang, E. Cu Nanoclusters with aggregation induced emission enhancement. *Small* **9**, 3873-3879 (2013).

S3. Huang, N. M. *et al.* γ-ray assisted synthesis of silver nanoparticles in chitosan solution and the antibacterial properties. *Chem. Eng. J.* **155**, 499-507 (2009).

S4. Nguyen, N. T. *et al.* Microwave-assisted synthesis of silver nanoparticles using chitosan: a novel approach. *Mater. Manuf. Processes* **29**, 418-421 (2014).

S5. Sambhy, V. *et al.* Silver bromide nanoparticle/polymer composites: dual action tunable antimicrobial materials. *J. Am. Chem. Soc.* **128**, 9798-9808 (2006).
